# The Asymmetry Bias in *Me, We–Others* Distance Ratings. The Role of Social Stereotypes

**DOI:** 10.3389/fpsyg.2016.00050

**Published:** 2016-02-03

**Authors:** Maria Jarymowicz, Marta Kamińska-Feldman, Anna Szuster

**Affiliations:** Faculty of Psychology, University of WarsawWarsaw, Poland

**Keywords:** object's saliency, social comparisons, egocentric vs. allocentric asymmetry

Studies in cognitive and social psychology have revealed that the distance from X to Y may be estimated as shorter or longer than the same distance from Y to X. Results showed that this judgment depended on particular properties of compared objects and a reference point in social comparisons (Holyoak and Mah, 1982; Codol, 1987; Kamińska-Feldman, 1991, 2012; Arcuri and Serino, 1992; Hurtig et al., 1993; Hoorens, 1995; Otten and Van Der Pligt, 1996; Eiser et al., 2001).

A similar type of illusion—called the *asymmetry effect*—was found in studies concerning diverse objects, including numbers or geometrical figures (Rosch, 1975), countries (Tversky, 1977) or self—others comparisons (Codol, 1984, 1993). Moreover, analogous asymmetry was displayed in estimations of psychological similarities between objects as in ratings of physical distances between objects (Codol, 1984, 1985). Thus, the asymmetry effect seems to be a universal phenomenon.

The current focus concerns explications of the asymmetry effects in the self—others physical distance ratings. The main question raised is: what are the determinants of the asymmetry effects? We attempt to argue that the asymmetry in the self—other(s) distance ratings bias can be due to the self being a cognitive prototype in social perception or to the cognitive stereotypes of the other(s).

## Diverse explanations of the asymmetry bias and the egocentric asymmetry effect

Rosch (1975) showed that people perceived the same distance between two objects as different when one was a prototype and the other was a variant of the prototype. Her studies concerned geometric figures (regular or irregular) or numbers (round or not round). One of the compared stimuli was placed on a semicircular board and the participant was asked to place the other at a distance that reflected the “felt distance” between the stimuli. Results showed that, if the number 100 was placed in the center of the board, participants placed the number 103 closer than when the number 103 was located centrally and participants had to place the number 100. Thus, the distance between the same two numbers was perceived differently depending on which of the numbers was being compared to which: the prototypical to the non-prototypical or *vice versa*. According to Rosch, prototypes serve as reference points in the classification of objects. A prototypical object determines how the other objects are perceived.

The same phenomenon was found in social perception (Tversky, 1977). Tversky asked participants (American students) about similarities between different nations. Participants viewed, for example, Poland as more similar to the USSR than the USSR to Poland. According to Tversky, the results could be explained by the USSR (and not Poland) being the prototypical country among the communist states.

Codol (1987, 1993) used the same argument when studying the self as a prototype in social perception. He assumed, in line with many of his contemporaries (Kuiper, 1981; Srull and Gaelick, 1983; Markus et al., 1985), that the self plays the role of a reference point in social comparisons. Codol (1984) observed the so-called *egocentric asymmetry effect*: the other appeared more similar (less distant) to the self than *vice versa*. Moreover, data revealed a positive correlation between the degree of the self-prototypicality and the size of the egocentric asymmetry effect (Codol et al., 1989).

In our studies on intergroup comparisons, we requested that participants estimate distances between the in-group vs. the out-group members (with regard to their ethnicity, race, physical typicality, or sexual orientation). In the we—others comparisons, we found asymmetry similar to that found in the self—others comparisons; the out-group members were estimated as less distant from the in-group members than *vice versa* (Szuster-Zbrojewicz, 1993).

## The allocentric asymmetry effect and its determinants

Further studies revealed that, in some conditions, the self—others comparisons did not lead to egocentric asymmetry, but to reverse asymmetry effects, and that determinants of the latter could be dispositional or situational (Kamińska-Feldman, 1988, 1993, 2002; Serino, 1992). Karyłowski (1990) found that egocentric asymmetry appeared when he stimulated concentration on the self; it did not appear in control conditions. Based on his studies, Karyłowski found that, although self-representation had a privileged position as a habitual reference point in social perception, the asymmetry effect occurred only when this representation was accessible. Moreover, when Kamińska-Feldman (1993) stimulated concentration on the self or on the other, the asymmetry effects were opposite. In the second case, the same distance from the self to the other was estimated as smaller than the distance from the other to the self. Kamińska-Feldman (1994) called this type of bias the *allocentric asymmetry effect*, referring to the assumption formulated by Holyoak and Gordon (1983). The authors suggested that the more elaborate was the stereotype of the target compared to the self, the weaker was the function of the self as a reference point in social perception, and the greater was the likelihood that the direction of the asymmetry in the self—others comparison would be reversed. Taking into account the more general assumption of Tversky (1977), that the asymmetry bias was, in fact, due to the degree of saliency of compared objects, Kamińska-Feldman (1994) formulated the hypothesis that allocentric asymmetry was always displayed when the stereotype of the other was salient (for any cognitive or affective reason). Empirical data were coherent with this supposition (Kamińska-Feldman, 1997, 2002, 2012; Kamińska-Feldman and Jarymowicz, 2006).

## The measurement of the asymmetry in ratings of physical distances between objects

Codol (1985) showed coherence between measures of the asymmetry effect in the self—others comparisons that referred to (1) estimates of the psychological similarity (measured by Tversky), and (2) the physical distance between objects (used by Rosch). In the latter case, participants were requested to estimate a distance from the self to the other, and the same distance from the other to the self. Codol found consistent results when objects were placed in a real space (in a room) or represented by drawings (paper techniques). Such a graphic technique was easy to use and allowed to precisely measure estimation errors.

To measure the asymmetry effects resulting from social stereotypes, we constructed a version of the graphic technique containing labels with first names typical of different nationalities (such as Antonio, Olaf, or Samuel). We assumed that names specific to particular nations would evoke generalized representations of those nations.

Participants were presented with drawings of a rectangle, where one point was labeled Me, and other points were labeled with diverse first names. They were requested to imagine that the points represented people in real space, and to estimate distances between some of the labels. In one case, they estimated the distance from Me to X—one representative of a particular nation. In the other case, they estimated the identical distance from Y—another representative of the same nation—to Me. For instance, one question could refer to a distance from Me to Boris, and another one to a distance from Nikita to Me. As a result, the difference between two such estimations indicated a direction and a size of the asymmetry effect in the self—Russians comparisons. The positive value of such an index demonstrated egocentric asymmetry, whereas the negative value demonstrated allocentric asymmetry.

## Studies on the determinants of allocentric asymmetry in objects' physical distance ratings

In some of our studies, participants estimated distances between themselves and the physically salient other with regard to race or physical disability (Jarymowicz, 2006). In the first case, we used pairs of Asian vs. European first names (like Chinese Ning and Cheng vs. Czechs Zdenek and Vaclav). In the second case, participants read a short story about a group of young people with one boy in a wheelchair. In each of these two types of studies, participants showed the egocentric asymmetry effect in the self—other Europeans ratings, and the self—physically typical other comparisons, while in the self—Asians and the self—disabled other distances ratings, they displayed allocentric asymmetry. We considered the latter pattern of the results due to social saliency (and lack of habituation in respect to people with different physical attributes).

Another type of study was related to others who, although physically similar to the Poles, were connected by social stereotypes (Jarymowicz, 2006). In Poland, this often concerns people of different sexual orientations, as well as different ethnic minorities. In each study conducted among heterosexual participants, we found the same effects: egocentric asymmetry in comparisons of the self—heterosexual other, and allocentric asymmetry in comparisons of the self—homosexual other. Similar were the results referring to diverse minorities and, especially, to the Poles—Jews comparisons (Kamińska-Feldman and Jarymowicz, 2006). Stereotypes of Jews are still salient, even among young Poles. Before World War II, the Jewish population in Poland was very large (mostly traditional and often orthodox); however, after the Holocaust, it dramatically decreased (and assimilated to Polish culture). Thus, today's distance toward Jews is due not to personal or social experience, but to stereotypes transmitted by the elder generation. In all data gathered in the course of these studies, we found the same pattern: in the self or we vs. other Europeans distance ratings, participants displayed the ego/ethnocentric asymmetry bias. However, in the self or we vs. Jews comparisons, participants displayed the opposite effect: the so-called *Judocentric asymmetry*.

We found evidence that supported the assumption that allocentric asymmetry was due to social stereotypes. A series of studies done by Grzesiak-Feldman (2006) showed a clear relationship between *Judocentric asymmetry* and stereotypical beliefs about Jews. We also found correlations between the same asymmetry effect and (1) an overestimation of the Jewish population in the contemporary world, and (2) the Implicit Association effect (Greenwald et al., 1998) in reaction to first names denoting Poles and Jews, which we considered a manifestation of automatic in-group favoritism and out-group discrimination (Jarymowicz, 2006).

## When does the asymmetry in social comparisons disappear?

The asymmetry bias was discovered more than 40 years ago as a particular kind of purely cognitive illusion. However, studies in social psychology showed that the perception of distances between individuals and groups could be due to motivational factors interfering with cognitive processes in such ways that the self—others distances ratings could be biased. This type of social cognition might be due to social stereotypes understood as rigid cognitive schemata with strong affective connotations (Jarymowicz, 2001). Thus, we expected the allocentric asymmetry effect to be significantly reduced among people without such specific stereotypes. Data have supported such a supposition. In a series of studies, in each group of participants, we found people who (in a given experimental condition) did not display the allocentric asymmetry effect. At the same time, the correlative data showed that these participants displayed a higher level of diverse manifestations of openness to people of different cultures, respect for standards of tolerance and values such as intellectual autonomy and social bonds (Schwartz, 1992, 1994; Kamińska-Feldman, 2002, 2012; Jarymowicz, 2008).

## Conclusions

We wanted to recall the diverse manifestations of the asymmetry bias and hypotheses concerning its nature. In reference to the empirical data, we pointed out that, in the self—others comparisons, the bias was displayed not only if the self was a prototype in social perception (which led to egocentric asymmetry), but also when the other had stereotypical cognitive representation (which led to allocentric asymmetry). The measurement of diverse forms of asymmetry bias could be a useful tool in studies on social stereotypes and relationships.

## Author contributions

When determining authorship the following criteria should be observed: MJ (1) Substantial contributions to the conception or design of the work; or the acquisition, analysis, or interpretation of data for the work–40%, (2) Drafting the work or revising it critically for important intellectual content–60%, and (3) Final approval of the version to be published–50%. AS (1) Substantial contributions to the conception or design of the work; or the acquisition, analysis, or interpretation of data for the work–20%, (2) Drafting the work or revising it critically for important intellectual content–40%, and (3) Final approval of the version to be published–50%. MK (1) Substantial contributions to the conception or design of the work; or the acquisition, analysis, or interpretation of data for the work 40%, (2) Drafting the work or revising it critically for important intellectual content–0%, and (3) Final approval of the version to be published.

### Conflict of interest statement

The authors declare that the research was conducted in the absence of any commercial or financial relationships that could be construed as a potential conflict of interest.
